# Evaluating Aboriginal and Torres Strait Islander Social and Emotional Wellbeing services: A collective case study in Far North Queensland

**DOI:** 10.1177/00048674241242935

**Published:** 2024-04-08

**Authors:** Mary Anne Furst, Tina McDonald, Janya McCalman, Jose Salinas-Perez, Ruth Fagan, Anita Lee Hong, Merrissa Nona, Vicki Saunders, Luis Salvador-Carulla

**Affiliations:** 1 Mental Health Policy Unit, Health Research Institute, University of Canberra, Bruce, ACT, Australia; 2 Jawun Research Centre, Office of Indigenous Engagement, CQUniversity, Cairns, QLD, Australia; 3 Department of Quantitative Methods, Universidad Loyola Andalucía, Seville, Spain; 4 Gurriny Yealamucka Health Service Aboriginal Corporation, Yarrabah, QLD, Australia; 5 Deadly Inspiring Youth Doing Good (DIYDG) Aboriginal and Torres Strait Islander Corporation, Cairns, QLD, Australia; 6 Menzies Centre for Health Policy, School of Public Health, The University of Sydney, Sydney, NSW, Australia; 7 National Centre for Epidemiology and Population Health (NCEPH), College of Health & Medicine, Australian National University, Canberra, ACT, Australia

**Keywords:** Social and emotional wellbeing, health service mapping, mental health systems

## Abstract

**Background:**

Access to a coordinated range of strengths-based, culturally appropriate community-led primary mental health and Social and Emotional Wellbeing services is critical to the mental health and wellbeing of young Aboriginal and Torres Strait Islander people, and is a policy commitment of the Australian government. However, complex and fragmented service networks and a lack of standardised service data are barriers in identifying what services are available and what care they provide.

**Method:**

A standardised service classification tool was used to assess the availability and characteristics of Social and Emotional Wellbeing services for young Aboriginal and Torres Strait Islander people in two regions in Queensland, Australia.

**Results:**

We identified a complex pattern of service availability and gaps in service provision. Non-Indigenous non-governmental organisations provided a significant proportion of services, particularly ‘upstream’ support, while Aboriginal Community Controlled Organisations were more likely to provide ‘downstream’ crisis type care. Most services provided by the public sector were through Child Safety and Youth Justice departments.

**Conclusions:**

Our findings demonstrate the complexity of current networks, and show that non-Indigenous organisations are disproportionately influential in the care received by young Aboriginal and Torres Strait Islander people, despite community goals of self-determination, and government commitment to increasing capacity of Aboriginal Community Controlled Organisations to support their local communities. These findings can be used to support decision making and planning.

## Introduction

Analysis of mental health and wellbeing service provision in Aboriginal and Torres Strait Islander communities must consider the core differences between Aboriginal and Torres Strait Islander concepts of health, and those defined by Western models of healthcare. For Australian Aboriginal and Torres Strait Islander peoples the Social and Emotional Wellbeing (SEWB) model is ‘a multidimensional concept of health’ (Gee et al., 2014), whose guiding principles include a holistic view of wellbeing, a strengths-based approach, centrality of family and kinship, self-determination and recognition of the experience of intergenerational trauma. These principles have been affirmed in a succession of Australian federal and state strategic documents (Department of Health and Aged Care, Australian Government, 2017, 2021; Department of the Prime Minister and Cabinet, 2017; Indigenous Allied Health Australia, n.d.) The Gayaa Dhuwi (Proud Spirit) Declaration asserts that ‘Culturally valid understandings must shape the provision of services and must guide assessment, care and management of Aboriginal and Torres Strait Islander peoples’ health problems generally, and mental health problems in particular’ (www.gayaadhuwi.org.au). However, the current health service network is complex, difficult to navigate and underutilised by young Aboriginal and Torres Strait Islander people (Azzopardi et al., 2018). The need for more data and better data systems to provide more informed policy and planning are the cornerstones of recent policy frameworks (Department of Health and Aged Care, Australian Government, 2020; Department of the Prime Minister and Cabinet, 2017).

A whole systems (Stansfield et al., 2020), or health ecosystems research (HER) approach (Furst et al., 2021), taking into account all sectors providing SEWB support can describe the total service availability in the local system, and illuminate the relationships between services. This requires mapping the service landscape to identify all the services available and what they are doing. A multisectoral service classification system that allows service description across sectors and comparison across divergent territories (i.e. urban and rural), and that can do so without the ambiguity inherent in the health services research is needed (Castelpietra et al., 2020).

The service classification system, Description and Evaluation of Services and DirectoriEs (DESDE; Salvador-Carulla et al., 2013), provides a standardised method of describing and classifying services in all sectors. It has mapped service provision at the local level in 35 countries across a range of sectors – and in a range of settings – rural, remote and urban, including areas with high Aboriginal and Torres Strait Islander populations, such as the Kimberley region and Far West New South Wales (Van Spijker et al., 2019). Integrated Atlases of Care based on the DESDE system have been developed in over a third of the health regions (Primary Health Networks) in Australia. DESDE has demonstrated its usability for disambiguating complex care provision in mental health (Gutierrez-Colosia et al., 2022).

This study aims to demonstrate the use of DESDE and a HER approach to describe the pattern of SEWB and mental health service provision for Aboriginal and Torres Strait Islander young people aged 5–18 years living in Cairns and in the discrete Aboriginal community of Yarrabah in Far North Queensland. Young people and service providers in Yarrabah have recently identified several areas of unmet needs, including availability of information about mental health, safe points of contact, access to youth facilities, support for recovery and early intervention (McCalman et al., 2023).

The Integrated Atlases of Social and Emotional Wellbeing of children and youth in Yarrabah and in Cairns (Furst et al., 2022, n.d.), using DESDE, comprise the service mapping component of the Systems Integration to Promote the Mental Health of Aboriginal and Torres Strait Islander Children and Youth research project (SIP) conducted through the Jawun Research Centre, Central Queensland University (CQU) (McCalman et al., 2020). SIP aims to ‘conceptualise, co-design and evaluate community-driven systems-level integration to promote the mental health and wellbeing of Indigenous school-aged children and youth’. Research for these Atlases was conducted by research teams from the Mental Health Policy Unit (formerly at Australian National University and now at Health Research Institute, University of Canberra), and Jawun Research Centre at CQU collaboratively with the SIP community-based research partners. In Yarrabah the SIP research partner is Gurriny Yealamucka Health Service, an Aboriginal and Torres Strait Islander community-controlled primary healthcare service. In Cairns, the research partner is Deadly Inspiring Youth Doing Good (DIYDG), an Aboriginal and Torres Strait Islander community-controlled youth empowerment organisation.

## Study design and study areas

Ecological, comparative and crosssectional study of one urban and one rural remote region in Far North Queensland.

### Yarrabah

Yarrabah, located 60 km east of Cairns in Far North Queensland, is Australia’s largest discrete rural Aboriginal community. Modern-day Yarrabah is on the traditional country of Gunggandji and Yidinji people, home to both their descendants, and to the descendants of other Aboriginal and Torres Strait Islander nations forcibly relocated following the establishment of an Anglican mission in Yarrabah in the 19th century. Yarrabah’s Shire Council runs a special local government area, managed under a Deed of Grant in Trust. Leaders from 15 community-based organisations in Yarrabah comprise the Yarrabah Leaders Forum, which acts as a collective for the benefit of the community, based on six overarching principles including safety, sustainability, health, supportive foundations and opportunity, including for employment.

In all, 96% of Yarrabah’s population of just over 2500 people identify as Aboriginal or Torres Strait Islander according to the 2016 census, almost half (43%) of whom are under the age of 20 years (Yarrabah’s total population figure is disputed, with alternative data collected by Queensland Police Service and Gurriny Yealamucka Health Services suggesting that it is closer to 4500). Yarrabah’s population experiences a level of social and economic disadvantage almost double that of the Australian average (Torrens University, n.d.). Almost half (47.3%) of children live in low-income families compared with the Australian average of 10.7%. Housing stock is inadequate, with 65.2% of people living in crowded dwellings (compared with the national average of 7.1%), and access to the Internet is lower than average (48.2%/39.2% compared with 80.2%). Despite these disadvantages, 100% of children aged 5 years are immunised and the rate of children enrolled in preschool education and of young people still in full-time education at age 16, though lower than the national average, is rated ‘good’. Local people derive strength from their connections with Country and culture and 92% of adults living in Yarrabah feel they can get support in times of crisis from people outside their immediate household.

Our community research partner in Yarrabah was Gurriny Yealamucka Health Service Aboriginal Corporation. Established in 2000 and taking full control of primary healthcare service delivery for Yarrabah in 2014, it was the first community-controlled health organisation in Australia to deliver primary health services in a discrete Aboriginal Community. Its workforce, comprised 70% of locals, delivers a range of culturally appropriate clinical and social healthcare services to the Yarrabah population. Gurriny and the Shire Council are major employers in Yarrabah.

### Cairns

Cairns is a city of 169,312 according to the 2021 census, 11.8% of whom identify as Aboriginal or Torres Strait Islander. The traditional owners of the land are the Gimuy Walubara Yidinji people, who continue to claim native title rights. The discovery of gold in the region in the late 19th century led to the establishment of a British settlement. Cairns is the commercial centre for Far North Queensland and Cape York regions, and a major domestic and international tourist destination. The Cairns and Hinterland Hospital and Health Service (HHS) area of Queensland Health covers 142,900 sq km and includes Yarrabah. It is responsible for providing hospital services to the approximately 253,000 people across this region. The Aboriginal Community Controlled Health Service providing primary care in the Cairns region is Wuchopperen Health Service Limited.

Like Yarrabah, the Aboriginal and Torres Strait Islander population in Cairns is also relatively young: 44% of its population is under 20 years of age. Aboriginal and Torres Strait Islander people in Cairns are also significantly more likely than the general population to live in crowded dwellings (22.5% compared with 7.1%). The percentage of children living in low-income families is 16.2%, and 82.3% of people have Internet access. Rates of childhood immunisation (95%) and of preschool enrolment in Cairns are also high; and 80% of young people are still in full-time education at the age of 16 years (Torrens University, n.d.). However these figures represent the total population, almost 90% of whom are not Aboriginal and Torres Strait Islander.

Deadly Inspiring Youth Doing Good (DIYDG) was the community research partner in Cairns. Founded in 2016 by Aboriginal and Torres Strait Islander young people, DIYDG is a youth led, not-for-profit Aboriginal and Torres Strait Islander Corporation. With a culturally diverse group of staff and volunteers, DIYDG’s mission is to ‘ inspire, equip and empower young people to take action and change the world’. DIYDG partners with a number of organisations and individuals in the Cairns region to provide a range of youth leadership and support programmes following principles of Aboriginal and Torres Strait Islander empowerment.

## Instrument

Data were collected and analysed using the Description and Evaluation of Services and DirectoriEs (DESDE), an internationally validated (Salvador-Carulla et al., 2013) instrument for the standard description and coding of services (Salvador-Carulla et al., 2006). DESDE classifies the type of care provided by individual care teams within each service, across multiple axes: target population (age, specific population group and diagnosis/reason for using service), the main type of care (MTC) the service provides and other relevant information – e.g., whether the service has stable ongoing funding. We have adapted DESDE to include the classification of services targeting whole populations or subpopulations at risk by the use of an additional qualifying code. Individual care teams within organisations are described as Basic Stable Inputs of Care (BSICs). The main type of care each team delivers is classified according to six main branches of care types (Residential, Outpatient [used here to include all centre-based or outreach services available in the community not included in the other categories, and not only clinical services], Day Care, Self-help and Voluntary Care, Information and Assessment, and Accessibility to services); and to characteristics such as acuity, mobility and intensity of service provision. The use of common units of analysis addresses methodological barriers in health services research related to terminological ambiguity (Gutierrez-Colosia et al., 2022) and commensurability bias (where use of different units of analysis does not provide like for like comparison).

Full time equivalents (FTEs) of the workforce providing direct care or support were collected where possible.

### Ethics

Ethical approvals were submitted by the CQU research team and granted as per the following:

- Central Queensland University (0000021644).
- Queensland Education Research Inventory (550/27/2319).
- Queensland Health, Cairns Hinterland Hospital Health Service (1557 / 04458-2021).

### Inclusion criteria

Services were included in the study if they met the following criteria:

- Target children, adolescents or young adults aged 5–18 years or their families/carers.
- Support the social and emotional wellbeing of Aboriginal and Torres Strait Islander children and/or youth in the region.
- Provide direct care or support to the target population.
- Located within the Cairns and Yarrabah regions.
- Do not require significant out-of-pocket expenses or membership of a fully private insurance scheme.

### Data collection

Data were collected between July 2021 and February 2022. All eligible services were identified and contacted by the CQU research team in consultation with the research partners. Requests for interviews were made by phone and email. Interviews with managers of participating services were conducted by CQU and UC research teams face to face, by phone or by Zoom Video, and were audio recorded, with written consent from interviewees. Recordings were stored electronically in line with CQU data management protocols. Interviewees were sent summaries of the interview for validation.

### Data analysis

Data from the interviews were entered into electronic spreadsheets, and each MTC provided by a care team was allocated a code by the UC team following analysis of its characteristics, according to DESDE criteria.

Availability is defined in this study as a service being operable upon demand to perform its designated or required function. Availability of each MTC in both regions was calculated per 100,000 people under 18 years identified as Aboriginal or Torres Strait Islander, according to the six DESDE domains as above to type of service provider, non-government organisation (NGO)/Aboriginal and Torres Strait Islander Community Controlled Organisation (ACCO)/public sector, age group and diagnosis/reason for using service. Workforce was analysed according to occupational type and FTE.

## Results

In Yarrabah, 10 organisations providing 23 BSICs (care teams), delivering 24 MTC were identified; 18 of these MTC were youth specific. A total of 16 organisations were identified in Cairns, providing 43 care teams (BSICs) delivering 48 MTC; 43 of these MTC were youth specific.

### Type of organisation delivering support

**Figure 1.**
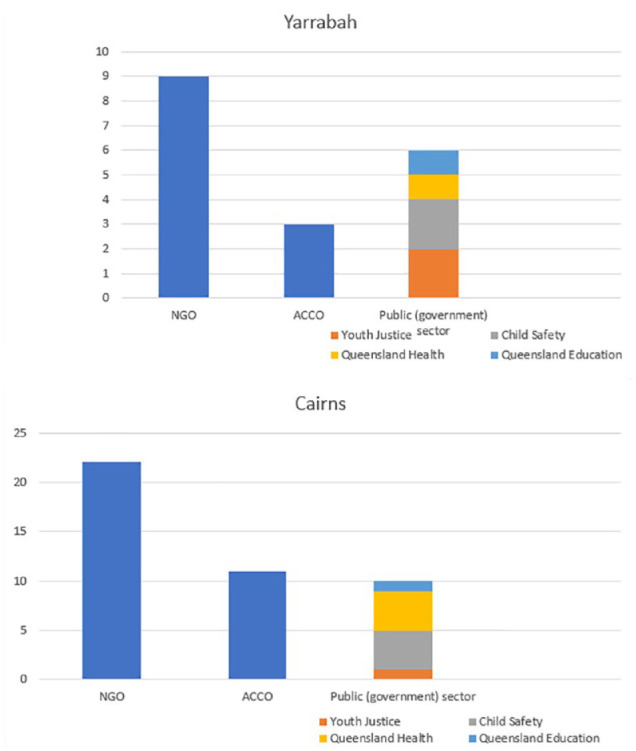
Number and type of organisation delivering services to children and youth in Yarrabah (top) and Cairns (below).

NGOs were by far the largest organisational type delivering youth-specific services in Yarrabah (providing 50% of services), followed by the public sector (33%), particularly child protection and youth justice services (22.2% of all services) and finally ACCOs, who delivered 16.6% of services. In Cairns, service provision was similarly dominated by the NGO sector (51%), the public sector 23.3% (child protection and youth justice services providing 11.6%), with a slightly higher proportion of services delivered by ACCOs (25.6%) (Figure 1).

**Figure 2.**
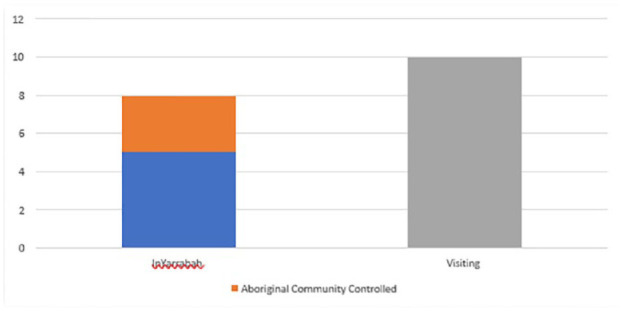
Proportion of services available to young people in Yarrabah according to whether visiting or based in community.

Fewer than half (44.4%) of the services available to youth in Yarrabah were provided by organisations based in Yarrabah, the remainder coming from organisations visiting from outside the community, predominantly Cairns. Of those services located in Yarrabah, fewer than half (37.5%) were provided by ACCOs, with the remainder provided by NGOs or the public sector (Figure 2). In Cairns, all services were located within the Cairns region.

### Distribution of MTC

**Figure 3.**
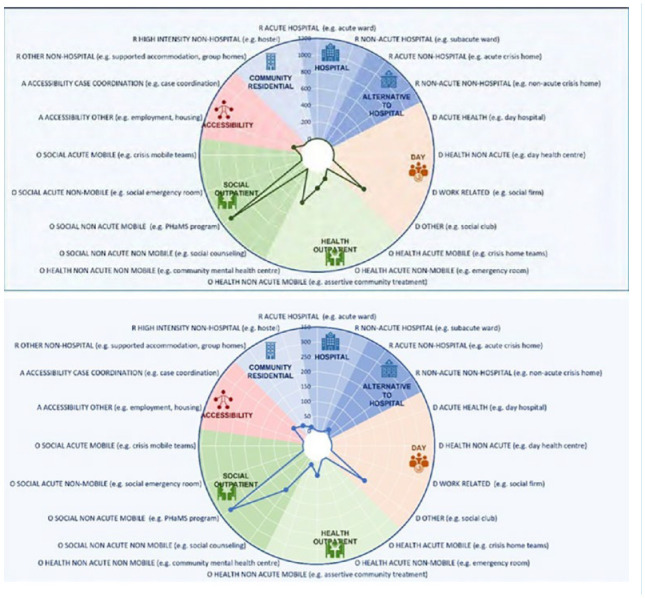
Distribution of Main types of Care: (top) Yarrabah; (bottom) Cairns.

Each of the 21 points on the radius of the diagram in Figure 3 represents the number of MTC in the relevant care category per 100,000 population under 18 years.

The overall pattern of care is similar in both regions, although Yarrabah lacks residential care and self help/volunteer run services, both of which are available in the larger region of Cairns. Services providing non-health-related outreach support were the most common type of service in both regions, with gaps in work- and education-related day services, accessibility services and residential care of all types. There was no acute youth residential mental health care available in either region, although a subacute residential youth mental health unit in Cairns sometimes provided acute support if needed.

### Service availability according to target age group

**Figure 4.**
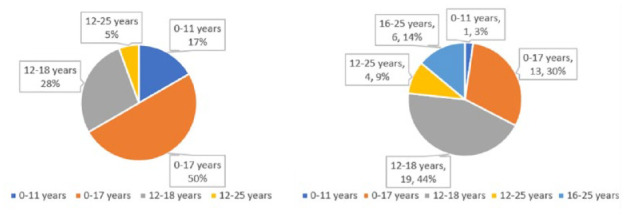
Service availability according to target age group: (L) Yarrabah; (R) Cairns.

The most common target population of services for youth in Cairns was teenagers aged 12–18 years, while in Yarrabah it was children and adolescents aged 0–17 years (Figure 4). There were fewer services specifically for younger children in both regions.

### Service availability according to type of service provider and reason for using service

**Figure 5.**
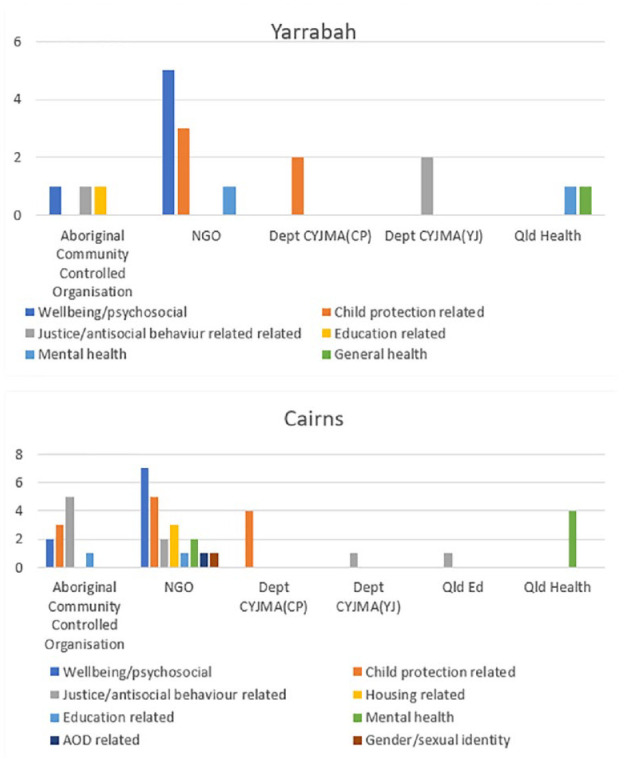
Types of service available according to type of provider organisation (top: Yarrabah; bottom: Cairns). NGO: non-governmental organisation; CYJMA: Department of Children, Youth Justice and Multicultural Affairs; CP: child protection; YJ: youth justice; QLD Ed: Queensland Department of Education. *Note.* since May 2023, the Department of Child Safety, Seniors and Disability Services and the Department of Youth Justice, Employment, Small Business and Training have been, respectively, responsible for child safety and youth justice in Queensland.

The type of care according to reason for using service delivered by each type of provider organisation (ACCO, NGO, public sector) was analysed (Figure 5).

In Yarrabah, ACCOs provided a roughly equal mix of general psychosocial/wellbeing (i.e. preventive) type support, child safety-related services and education-related support. NGOs provided primarily general wellbeing (preventive) type support, followed by services related to child protection. Mental health-related services specifically for young people were provided by the NGO sector and Queensland Health.

In Cairns, ACCOs were most likely to deliver justice-related support. They provided services to all age groups except specifically to the 0–11 year age group.

### Workforce

Workforce data (Table 1) should be viewed with caution as we were unable to get comprehensive data from many services. In addition, some of the positions labelled below may have been occupied by Aboriginal and Torres Strait Islander peoples but not identified as such. Most teams in both regions were small. Workers identified as Aboriginal comprised 6% of the workforce providing support to young Aboriginal and Torres Strait Islander people in Yarrabah and 5% of the workforce in Cairns.

**Table 1.** Size of teams providing support.

Size of team (FTE)	Number of services	
	Yarrabah	Cairns
Very small (<1 or 1 FTE)	0	5
Small (1/1–5.9 FTE)	16	11
Medium (6–20 FTE)	4	6
Large (20+ FTE)	0	0

FTE: full time equivalents.

## Discussion

To our knowledge, this study provides the first standard comparative analysis of local support and care systems following a national Aboriginal model of health (SEWB). Using DESDE, we have identified and described SEWB services across all relevant sectors in urban Cairns and the adjacent discrete Aboriginal community of Yarrabah. We have identified patterns of service provision and gaps in service availability, and conducted a preliminary analysis of the workforce. The data corroborate the narratives of young people and local service providers in Yarrabah of limited service availability, and raised questions about the balance of service provision, funding and accountability, and the scope of services provided by ACCOs.

The dominance of the non-Indigenous NGO sector and large footprint of the youth justice and child protection sector in Indigenous youth services raises questions around the capacity of the system to deliver on policy commitment to Aboriginal and Torres Strait Islander leadership and models of support; the extent of engagement of Aboriginal and Torres Strait Islander people in service governance and decision making; how accountable non-Indigenous services are for the delivery and outcomes of culturally appropriate services; the number of Aboriginal and Torres Strait Islander people employed to provide culturally competent support; and how connected service providers are with the communities to whom they are funded to deliver services. For example, the research team was told of an NGO funded to deliver counselling to school children in Yarrabah, which visited the school only on Fridays, the day of lowest school attendance, potentially missing those children most in need of support. Further research is needed to identify how organisations are ensuring that their services are culturally appropriate, locally relevant and that their outcomes have cultural validity.

Although Aboriginal SEWB models of health are intrinsically holistic, located in positive and protective aspects of social and emotional wellbeing and thus closely aligned with ‘upstream’ preventive models of care, we found that non-Indigenous NGOs were providing most of this type of care, with ACCOs more likely to be funded to provide high-level support ‘downstream’, to children and families already in crisis. Our detailed analysis of service characteristics revealed ambiguity and complexity in the system not apparent through a simple analysis based on the official names of services, and is essential in identifying the real drivers of the system. Further analysis of funding networks is required to identify how, or to what extent, the Key Performance Indicators of non-Indigenous funding organisations affect the capacity of recipient Aboriginal and Torres Strait Islander services to deliver culturally appropriate care based on an SEWB model.

Key gaps in service availability were identified, including in community-based residential support. Young Aboriginal and Torres Strait Islander people requiring acute clinical residential support must either travel to the acute mental health unit in Townsville, some 350 km away from their communities, or be admitted to a general ward in Cairns. The sub-acute residential mental health service in Cairns was not identified as being in the referral pathways of any service interviewed. With 75% of the young people accessing a youth housing NGO identifying as Aboriginal or Torres Strait Islander, there is clearly also a need for Aboriginal and Torres Strait Islander-led accommodation options to assist young people regain and maintain vital links with their community and culture. Gaps were also identified in education and employment services despite the prominence of these sectors in policy. Short-term programmes such as those often delivered in schools are not included in the DESDE methodology due to their inherent lack of temporal and organisational stability.

Workforce data was incomplete within the data systems available to this research and should be viewed with caution: not all services could provide information disaggregated by FTE or by occupation. However, some initial concerning observations include the high proportion of non-Indigenous staff, and an imbalance in the proportion of non-Indigenous staff employed in roles at higher levels of decision making, such as tertiary trained health and social professionals. This has implications for Aboriginal and Torres Strait Islander input into decision making, as well as for the availability of Aboriginal and Torres Strait Islander role models in skilled health professional positions.

This is the first application of DESDE in a specifically Aboriginal and Torres Strait Islander context (Salinas-Perez et al., 2020). We were able to identify and classify each care team and the MTC it provides. A separate section of the DESDE (Section D) allowed identification of models and goals of care, critical to identify disjunction between the different levels of organisation. The data in Integrated Atlases can provide the basis for additional analysis such as financing flows, social network analyses and measures of specific domains of SWEB. The complexity of systems of SEWB service delivery make these additional analyses critical to a full understanding of the system.

Indigenous concepts of wellbeing include dimensions and attributes not recognised in Western categorising tools. SEWB services and their target populations extend not just across a range of sectors, but also across the whole health and wellbeing spectrum: from those providing a general wellbeing/preventive or health promotion role at population or subpopulation level, to those providing clinical interventions for individuals with specific therapeutic needs. In this context, we adapted DESDE to include services targeting whole populations or subpopulations at risk through an additional qualifying code. In addition, the ‘Z’ section of the International Classification of Diseases (ICD), which classifies health service use according to broader psychosocial circumstances rather than to individual deficit was used to indicate broader target subpopulations. Nevertheless, other, more strengths-based coding systems should be investigated for use in future.

### Limitations

While we were able to include all SEWB services, without exception, in Yarrabah, this was not feasible in Cairns, where services were identified by the CQU research team based on extensive local knowledge. Relevant services in the referral pathways of interviewed services were also identified at interview. This study uses a heuristics approach, whereby a ‘Satisficing’ threshold is reached, and the collection of further information confers no additional benefit (Djulbegovic et al., 2018). This approach is useful in analysis of complex situations such as this and allows experts’ consideration in such analysis (Marewski and Gigerenzer, 2012; Wolpert and Rutter, 2018).

Some services provided for young people up to the age of 18 years also included those up to 25 years in their target population.

Services requiring a significant out of pocket cost were not included in this report. The inclusion of private providers in the mapping of universally accessible services could distort the results. Service information was collected through interviews with service managers. Some information may not be accurate and should be objectively confirmed.

We acknowledge that there may be services outside the area and not included that may also be used by people in Yarrabah or Cairns.

These results provide a baseline of workforce capacity from which analyses of future need can be monitored. However, the comprehensiveness and accuracy of workforce capacity data are limited by the availability of these data and by the lack of reliable and standardised data to categorise the various roles, particularly in the non-registered professional workforce. In particular, it is important to note that the particular qualifications, experience and knowledge provided by Aboriginal and Torres Strait Islander people in the workforce, the work that they undertake in these roles and its significance to Aboriginal and Torres Strait Islander concepts of wellbeing (Garvey et al., 2021) are not properly acknowledged or included in the workforce classification descriptions. Future steps must include their incorporation into descriptions of workforce capacity.

## Conclusion

Using DESDE, this study has shown that the SEWB service delivery system for Aboriginal and Torres Strait Islander youth in these regions is highly complex with gaps in key areas. ACCOs play key roles within their communities in service provision; however, funding patterns direct their service provision primarily downstream rather than towards the preventive wellbeing space more closely connected to the cultural domains of SEWB. Although data on workforce are incomplete, the overall picture appears to show that Aboriginal and Torres Strait Islander people are underrepresented in the workforce.

Numerous policy documents over many years have recognised and reiterated the impact of colonisation and the need for community driven, culturally competent support for the health and wellbeing of Aboriginal and Torres Strait Islander children and young people. The findings of this study provide evidence about the current availability of services which can be used to support decision making and planning in the pursuit of these.
